# Items

**Published:** 1899-11

**Authors:** 


### ITEMS.

### A SCOFFER.

### THE CHRISTIAN SCIENTIST HAD GOOD REASONS FOR BELIEVING HER PATIENT HAD NO FAITH.

(*From The New-Orleans Times-Democrat.*)

"I WITNESSED a decidedly picturesque incident coming in from Chattanooga yesterday," said a guest at one of the hotels last night. "There were four of us at the end of the car—a business man from Houston, two women from Wilmington, Del., and myself, all middle aged, serious people, and naturally we drifted into conversation. Before long one of the women, who was a stout, matronly person, informed us that she was a christian science 'demonstrator,' and, needless to say, that started an interesting discussion. She gave us the usual nebulous 'explanation' of the cult, which no outsider has ever been able to understand, and then proceeded to describe a number of miraculous cures she claimed to have effected.

"The Houston man had said very little, but finally he remarked that he had been suffering for several years from a stiffness of the right ankle. 'You mean you think you have been suffering,' said the christian scientist. 'There is really nothing the matter with your ankle; in fact, it does not exist, except in your mind.' 'Well, it seems very real,' said the Houston man, 'and gives me a great deal of trouble. Do you think you could do it any good?' The Wilmington woman smiled in a pitying sort of way and replied that she could undoubtedly effect a cure. 'It may take some time to cleanse your mind of the delusion,' she said, 'and we can't begin too soon. Suppose I give you a treatment right now?' 'By all means, madame,' said the Texan, and the portly scientist closed her eyes and apparently took a short nap. The rest of us waited in solemn silence. In about ten minutes she shook herself and smiled blandly. 'Well, sir,' she said, 'you feel a good deal better now. The stiffness in your ankle is going away; going away; going away rapidily.' 'I am sorry to contradict you,' interrupted the Houston man, 'but it's just as bad as ever. If you'll listen you can hear it squeak.' He moved his foot on the floor, and, sure enough, it gave out a horrible creaking sound.

"The christian scientist turned pale. 'Good heavens!' she gasped, 'what is the matter with it anyhow?' 'Needs oiling, I think,' said the Texan. 'I forgot to mention that this foot is artificial.' The fat lady bounced to her feet and her double chins quivered with indignation. 'I am forced to believe,' she exclaimed dramatically, 'that you are no gentleman, sir! and a scoffer, sir! And I will bid you good day!' she said as she swept into the aisle. The two women retired to the next car, and the Texan and I laughed for an hour by the watch."

---

Faith Healers Indicted.—The grand jury of Porter County, Indiana lately returned indictments against Mrs. G. J. Smith, Mrs. M. L. Anderson and Mrs. Edward Smackles, converts of Dr. Dowie, the Chicago faith cure healer, charging them with being responsible for the death of the son of Henry Erne, who died without medical attendance, the only means adopted for his relief being the reciting of prayers. The grand jury of Lake County also began an investigation for two deaths attributed to criminal neglect by the practice of faith curing.

---

It is stated that the village of Findlay Lake, Chautauqua County, N. Y., needs a competent physician. A young man of professional ability would undoubtedly find this a valuable field.

It is quite probable also, that Chautauqua, N. Y., would prove an attractive place for an enterprising physician. In the summer it is visited by many thousand people from every part of the country, and the environment is an agreeable one.

